# Association of statin use with risk of Gleason score‐specific prostate cancer: A hospital‐based cohort study

**DOI:** 10.1002/cam4.2500

**Published:** 2019-10-08

**Authors:** Kai Wang, Travis A. Gerke, Xinguang Chen, Mattia Prosperi

**Affiliations:** ^1^ Department of Epidemiology University of Florida Gainesville Florida; ^2^ Department of Epidemiology Harvard T.H. Chan School of Public Health Boston Massachusetts; ^3^ Department of Cancer Epidemiology Moffitt Cancer Center Tampa Florida

**Keywords:** dose‐response, Gleason score, lipophilicity, prostate cancer, statins

## Abstract

**Background:**

Conflicting evidence suggests that statins act chemopreventively against prostate cancer (PCa). Whether the association of statin use with PCa risk is Gleason score‐dependent, time‐, dose‐respondent is not well studied.

**Methods:**

We conducted a cohort study at a tertiary hospital in the Southeastern US using longitudinal data of electronic medical records (EMR) from 1994 to 2016. Only cancer‐free men aged >18 years at baseline with follow‐up time of ≥12 months were included. Time‐dependent Cox proportional hazards regression was used to estimate adjusted hazard ratios (aHRs) with 95% confidence intervals (CIs).

**Results:**

Among 13 065 men, 2976 were diagnosed with PCa over median follow‐up of 6.6 years. Statin use was associated with lower risk of both Gleason low‐ (score <7: aHR, 0.85; 95% CI, 0.74‐0.96) and high‐grade PCa (score ≥7: aHR, 0.54; 95% CI, 0.42‐0.69). The protective association was observed only when statins had been used for a relatively longer duration (≥11 months) or higher dose (≥121 defined daily doses), and were more pronounced for PCa of higher Gleason score (<7: aHR, 0.85, 95% CI, 0.74‐0.96; 7 [3 + 4]: aHR, 0.62, 95% CI, 0.43‐0.90; 7 [4 + 3]: aHR, 0.49, 95% CI, 0.29‐0.82; 8: aHR, 0.60, 95% CI, 0.37‐0.96; 9‐10: aHR, 0.24, 95% CI, 0.11‐0.54). Lipophilic statins (aHR, 0.83; 95% CI, 0.72‐0.95) might be more protective than hydrophilic statins (aHR, 0.91, 95% CI, 0.63‐1.33) against PCa.

**Conclusion:**

Statin use might be associated with reduced PCa risk only when used for a relatively longer duration, and the risk reduction was higher for PCa of higher Gleason score.

## Graphical abstract

We found that statin use might be associated with reduced risk of both low‐ and high‐grade prostate cancer, but the reduced risk was observed only when statins had been used for a relatively longer duration or higher dose. Also, the statin‐related risk reduction was higher for prostate cancer of a higher Gleason score, and lipophilic statins might be more protective than hydrophilic statins against prostate cancer.

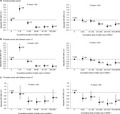

## 1 INTRODUCTION

Prostate cancer (PCa) is the most commonly diagnosed solid organ cancer and the second leading cause of cancer death among men in the US.^1^ PCa is often a slowly growing cancer with a long latency period of up to 15 to 20 years.^2^ Autopsy studies showed that a quarter of men in 40s and up to 40% of men aged 80 years or older harbor indolent local malignant lesions of the prostate.^3^ The high lifetime incidence and slow rate in PCa development make PCa an attractive target for chemoprevention.

Statins are a family of cholesterol‐lowering medications with documented effectiveness in preventing cardiovascular disease.^4^ The past 15 years have seen a dramatic increase in the prevalence of statin use, and statins are among the most prescribed medications in both the US and worldwide.^5^ Lowering serum and tissue cholesterol is thought to bring about a disruption in cellular lipid rafts, leading to reduced raft‐dependent signaling and cell proliferation.^6^ Therefore, statins may play a chemopreventive role in reducing the risk of carcinogenesis, including prostate carcinogenesis. Because PCa is highly incident and few preventive strategies currently exist, elucidation of a benefit of statins in preventing PCa could confer a considerable significance in public health. Clarifying the statin‐PCa association may also cast light to elucidate the steroid hormone mechanism connecting metabolic disorders with prostate carcinogenesis.^7^, ^8^

Statin use has been reported to be negatively correlated with advanced PCa risk,^9^, ^10^, ^11^ but evidence for the associations of statin use with overall PCa remains mixed^9^, ^10^, ^11^, ^12^, ^13^ and with Gleason score‐specific PCa is rare. Reasons for such a varying observation are not clarified but may result from a detection bias (“healthy user bias”).^14^, ^15^, ^16^ Because statin users are in a higher need of health services than those without using the medication, they may have more chances to take a prostate‐specific antigen (PSA) testing and thus are more likely to detect subclinical PCa. Therefore, a lack of statistical adjustment for PSA testing and PCa grade would considerably limit the study power on statins and PCa risk. Even after attempting to control for the potential healthy user bias, several other questions remain unclear: does the relationship between statin use and PCa risk have duration‐ and/or dose‐response properties; is statin use associated with PCa risk in a Gleason score‐specific manner; and does statin lipophilicity affect the association? To clarify these important questions, further studies on statin and PCa are needed.

Therefore, we used a large hospital‐based longitudinal dataset to examine the association of statin use with PCa risk and specific Gleason score at diagnosis. Further, we explored whether the relationship is duration‐ and/or dose‐respondent and statin lipophilicity‐dependent.

## 2 METHODS

### 2.1 Study population

A cohort study was conducted at a tertiary hospital affiliated to a university in the Southeastern US based on electronic medical records (EMR) data. This large‐scale health database was administered and provided by a professional Clinical and Translational Science Institute (CTSI) at the hospital and university, and collects and organizes information across all the university's clinical and research institutes located across the whole state. We included patients with at least one visit of the urologic clinic because of any prostatic conditions. International Classification of Disease, Ninth Revision (ICD‐9) codes (Table S1) were used to derive the data. For each included man, we then extracted all his medical records of previous and subsequent hospital visits throughout all medical departments (not limited to the urologic clinic). Data of demographics, lab results, medical diagnoses, medication prescriptions, and other clinical information were linked by a de‐identified patient ID. For each participant, the time of his first recorded hospital visit served as baseline and subsequent hospital visits served as follow‐ups. We only analyzed data measured before or at the time of PCa diagnosis. For men not diagnosed with PCa, the most recent hospital visit served as the time point of censoring. We excluded men 1) aged ≤18 years at baseline (n = 27) or 2) with a follow‐up duration of <12 months (n = 6993), resulting in the exclusion of 7020 men (4591 PCa and 2429 non‐PCa). We included a total of 13 065 men in the analysis, with the medical records ranging from November 1994 to January 2016.

This study has been performed in accordance with the Declaration of Helsinki. The research protocol was approved by the institutional review board at the University of Florida.

### 2.2 Assessment of statin exposure

We included all types of statins on the US market. These statins comprised of hydrophilic and lipophilic statins, with the former included rosuvastatin and pravastatin, and the latter included fluvastatin, lovastatin, simvastatin, atorvastatin, and cerivastatin.

We created statin use as a time‐dependent variable. Men were treated as statin nonusers up until the time when they were prescribed with statins for the first time, and as users thereafter for the remaining follow‐up time. Therefore, participants contributed the person‐time before their first statin prescription into the statin nonuser group, and after that time point into user group. We analyzed statin use in three time‐dependent variables: binary status (user or nonuser), cumulative duration of statin use, and cumulative dose of statin use. By analyzing cumulative duration and dose, we aimed to explore whether statins associate with the risk of PCa in a time‐ or dose‐response form. In a time‐dependent form, we created the cumulative duration by summing the months since the prior statin prescription for every follow‐up (hospital visit). We then categorized this variable by quintiles (1‐10, 11‐29, 30‐60, 61‐101, and 102‐239 months) among statin users throughout all follow‐ups. We then evaluated the cumulative dose of statin by units of defined daily dose (DDD).^17^ We used the DDD of simvastatin in 20‐mg formulation as the reference, and used the DDDs of the other statins to convert them to 20‐mg simvastatin‐equivalent doses. Likewise, cumulative dose was calculated by summing all DDDs for each follow‐up, and categorized by quintiles among statin users (5‐40, 41‐120, 121‐240, 241‐615, and 616‐38750 DDDs).

### 2.3 Outcome ascertainment

The outcome of interest was newly diagnosed PCa. PCa diagnosis was extracted from EMR database using ICD‐9 code for malignant neoplasm of prostate of 185 and carcinoma in situ of prostate of 233.4. We also derived the specific Gleason score for all PCa diagnosis. Due to the limited case number in some Gleason score categories (7 [3 + 4]: 259 cases; 7 [4 + 3]: 169 cases; ≥8:240 cases) and in order to have a better statistical power, in the main analysis, we merged the three categories of 7 (3 + 4), 7 (4 + 3), and ≥8 and referred to them totally as high‐grade PCa. PCa with Gleason score of <7 was referred to as low‐grade PCa.

### 2.4 Covariates

We also assessed potential confounders for the association between statin use and PCa risk, including age (years), race (white, black, and other), smoking status (current, former, and never), body mass index (BMI) (<18.5, 18.5 to <25, 25 to <30, and ≥30 kg/m^2^), PCa family history; history of diabetes, cardiovascular disease, chronic kidney disease, hyperlipidemia, hypertension, and benign prostatic disease; medication use of aspirin, angiotensin‐converting enzyme inhibitor, insulin, vitamin E/multivitamin, finasteride, metformin, testosterone, selenium; PSA level (<4, 4‐10, and >10 ng/mL) and cumulative times of PSA testing.

### 2.5 Statistical analysis

Characteristics of the study sample at baseline (time window of 1 month) were assessed using descriptive statistics, including median, interquartile range (IQR), and proportions. Chi‐squared test for categorical variables and Wilcoxon rank‐sum test for continuous variables were used to compare baseline characteristics of the cohort grouped by statin use throughout the study period. We estimated hazard ratios (HRs) and 95% confidence intervals (CI) for overall, low‐, and high‐grade PCa using time‐dependent Cox proportional hazards regression models with participants' age as the time metric. We evaluated statin use (use vs no use), cumulative duration of statin use (quintiles vs no use), and cumulative dose of statin use (quintiles vs no use) as time‐dependent variables, which were updated at the end of each calendar year during follow‐up. Time at risk began to accrue at baseline and ended on the date of PCa diagnosis or censoring, whichever occurred first. Linear trend of cumulative statin duration and dose in relation to PCa risk were examined by entering the quintiles of these variables in continuous form. We adjusted for baseline covariates with potential to confounder the statin‐PCa association (listed in Table 1). Diagnosis of cardiovascular disease (CVD), as a main clinical reason for statin prescription, together with cumulative number of PSA testing was entered as time‐dependent covariate. The proportional hazards assumption was tested by adding an interaction term of participant age and statin use to the Cox regression models. The term was not statistically significant by the likelihood ratio test, indicating no violation of the assumption.

**Table 1:** Baseline demographic and clinical characteristics of the cohort by statin use during study period

Characteristic	Entire cohort (n = 13 065)		Nonstatin user (n = 9226)		Statin users (n = 3839)		*P* value^a^
	No.	%	No.	%	No.	%	
Age, years							
Median	62		61		65		<.001
Interquartile range	53‐71		51‐70		57‐72		
Race							
White	6207	47.5	4308	46.7	1899	49.5	.004
Black	1057	8.1	710	7.7	347	9.0	.010
Other	324	2.5	260	2.8	64	1.7	<.001
Unknown	5477	41.9	3948	42.8	1529	39.8	.002
Smoking status							
Current smoker	602	4.6	395	4.3	207	5.4	.006
Former smoker	3105	23.8	2000	21.7	1105	28.8	<.001
Never smoke	2968	22.7	2265	24.5	703	18.3	<.001
Unknown	6390	48.9	4566	49.5	1824	47.5	.039
Body mass index, kg/m^2^							
<18.5	84	0.6	39	0.4	45	1.2	<.001
18.5 to <25	962	7.4	487	5.3	475	12.4	<.001
25 to <30	1338	10.2	571	6.2	767	20.0	<.001
≥30	1334	10.2	560	6.1	774	20.2	<.001
Unknown	9347	71.5	7569	82.0	1778	46.3	<.001
Prostate‐specific antigen, ng/mL							
<4	7206	55.2	5087	55.1	2119	55.2	.951
4‐10	963	7.4	710	7.7	253	6.6	.028
>10	324	2.5	261	2.8	63	1.6	<.001
Unknown	4572	35.0	3168	34.3	1404	36.6	.015
Family history of prostate cancer	225	1.7	183	2.0	42	1.1	<.001
Comorbidities							
Hypertension	4609	35.3	2739	29.7	1870	48.7	<.001
Hyperlipidemia	2972	22.8	1601	17.4	1371	35.7	<.001
Benign prostatic diseases	2790	21.4	1912	20.7	878	22.9	.006
Atherosclerotic cardiovascular disease	2579	19.7	1109	12.0	1470	38.3	<.001
Diabetes	1625	12.4	849	9.2	776	20.2	<.001
Chronic kidney disease	477	3.7	247	2.7	230	6.0	<.001
Histories of medication							
Aspirin	1738	13.3	632	6.9	1106	28.8	<0.001
ACE inhibitor	1115	8.5	400	4.3	715	18.6	<.001
Insulin	1102	8.4	476	5.2	626	16.3	<.001
Vitamin E/multivitamin	809	6.2	403	4.4	406	10.6	<.001
Finasteride	165	1.3	68	0.7	97	2.5	<.001
Metformin	135	1.0	56	0.6	79	2.1	<.001
Testosterone	14	0.1	7	0.1	7	0.2	.09
Selenium	6	0.1	2	0.02	4	0.1	.07^b^
Follow‐up time, years							
Median	6.6		6.0		8.3		<.001
Interquartile range	2.3‐11.5		1.9‐10.9		3.6‐12.7		

Abbreviation: ACE, angiotensin‐converting enzyme.

a Chi‐squared test or Wilcoxon rank‐sum test.

b Fisher's exact test.

We further stratified the analyses by low‐/high‐PCa Gleason grade (Gleason score <7 and ≥7), specific Gleason score (<7, 7 (3 + 4), 7 (4 + 3), 8, 9‐10), and statin lipophilicity. In the analysis of statin lipophilicity, statin users were classified by the following three categorical variables: only used lipophilic statins, only used hydrophilic statins, and used both lipophilic and hydrophilic statins.

### 2.6 Sensitivity analysis

We also stratified the analyses by baseline PSA level (<4 ng/mL, ≥4 ng/mL, and unknown), CVD diagnosis (yes/no), and combination of statin and aspirin use during follow‐up (no use of statins or aspirin, only statins, only aspirin, and both statins and aspirin).

We used R 3.1.1 (R Development Core Team 2014) for the data analysis. All *P* values were two‐tailed and *P* < .05 was statistically significant.

## 3 RESULTS

### 3.1 Characteristics of study participants

Among the 13 065 men included, median (IQR) age at baseline was 62 (53, 71) years. Over a total of 94 801.6 person‐years follow‐up (median 6.6 years), 2976 were diagnosed with PCa, among which 2308 were low‐grade (Gleason score <7) and 668 were high‐grade (Gleason score ≥7). Specifically, 259 were of Gleason score 7 (3 + 4), 169 were of 7 (4 + 3), 136 were of 8, and 104 were of 9‐10. Of the total sample, 3839 (29.4%, 3839/13065) used statins during the study period. Baseline characteristics by statin use throughout all follow‐ups are listed in Table 1.

### 3.2 Association of statin use with risk of overall PCa

**Figure 1:**
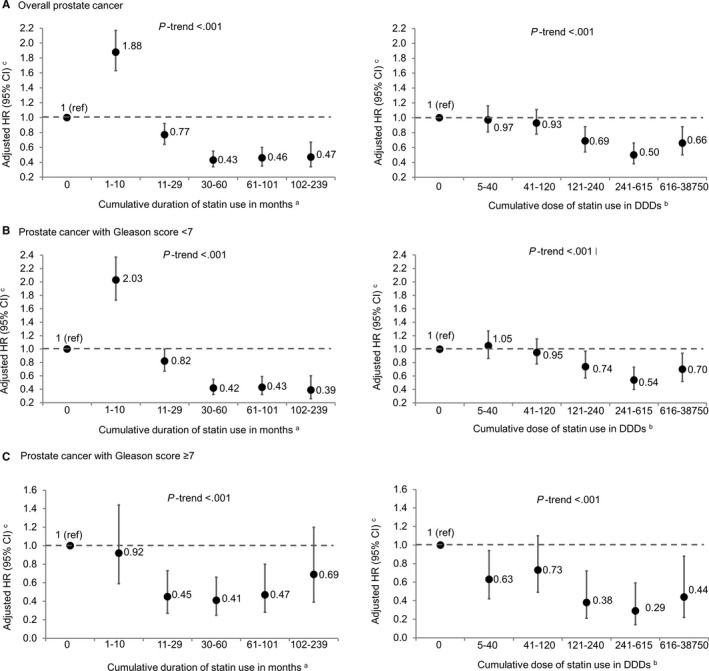
Risk of overall (A) and Gleason grade‐specific prostate cancer (B and C) by cumulative duration and dose of statin use. ^a^Statin users stratified by quintiles of cumulative duration of statin use during the study period. ^b^Statin users stratified by quintiles of cumulative dose of statin use during the study period. ^c^From time‐dependent Cox proportional hazards regression using age as time metric adjusting for race, family history of prostate cancer; baseline smoking status, body mass index, hypertension, hyperlipidemia, benign prostatic diseases, diabetes, chronic kidney disease, use of aspirin, angiotensin‐converting enzyme inhibitors, insulin, vitamin E/multivitamin, finasteride, metformin, testosterone supplement, selenium, prostate‐specific antigen level; as well as atherosclerotic cardiovascular disease and cumulative number of prostate‐specific antigen tests as time‐dependent variables

In the fully adjusted model, statin use was significantly associated with decreased risk of overall PCa (adjusted HR [aHR], 0.80; 95% CI, 0.71‐0.90), compared with no statin use. A decreasing pattern in PCa risk was observed with increasing cumulative duration (≥102 months: aHR, 0.47; 95% CI, 0.34‐0.67; *P*_trend_ < .001) and cumulative dose (≥616 DDDs: aHR, 0.66; 95% CI, 0.50‐0.88; *P*_trend_ < .001) of statin use. Of note, the protective association of statin with PCa was only observed with a relatively longer duration (≥11 months) or higher dose (≥121 DDDs). A short‐term statin use (1‐10 months) was observed to be associated with an increased PCa risk (aHR, 1.88; 95% CI, 1.63‐2.17) (Table 2; Figure 1).

**Table 2:** Association of statin use with risk of overall and Gleason grade‐specific prostate cancer

Statin exposure	Overall		Gleason 2‐6		Gleason 7‐10	
	No. of cases	HR (95% CI)^a^	No. of cases	HR (95% CI)^a^	No. of cases	HR (95% CI)^a^
No use of statins	2453	1.00	1868	1.00	585	1.00
Use of statins	523	0.80 (0.71‐0.90)	440	0.85 (0.74‐0.96)	83	0.54 (0.42‐0.69)
Cumulative duration, months						
Quint 1: 1‐10	233	1.88 (1.63‐2.17)	211	2.03 (1.73‐2.37)	22	0.92 (0.59‐1.44)
Quint 2: 11‐29	126	0.77 (0.64‐0.92)	110	0.82 (0.67‐1.00)	16	0.45 (0.27‐0.73)
Quint 3: 30‐60	72	0.43 (0.34‐0.55)	55	0.42 (0.32‐0.55)	17	0.41 (0.25‐0.66)
Quint 4: 61‐101	57	0.46 (0.35‐0.60)	42	0.43 (0.32‐0.59)	15	0.47 (0.28‐0.80)
Quint 5: 102‐239	35	0.47 (0.34‐0.67)	22	0.39 (0.26‐0.60)	13	0.69 (0.39‐1.20)
*P*_trend_		<0.001		<0.001		<0.001
Cumulative dose, DDDs^b^						
Quint 1: 5‐40	171	0.97 (0.81‐1.16)	144	1.05 (0.86‐1.27)	27	0.63 (0.42‐0.94)
Quint 2: 41‐120	166	0.93 (0.78‐1.11)	136	0.95 (0.78‐1.15)	30	0.73 (0.49‐1.10)
Quint 3: 121‐240	74	0.69 (0.54‐0.88)	64	0.74 (0.57‐0.97)	10	0.38 (0.21‐0.72)
Quint 4: 241‐615	56	0.50 (0.38‐0.66)	48	0.54 (0.40‐0.73)	8	0.29 (0.14‐0.59)
Quint 5: 616‐38750	56	0.66 (0.50‐0.88)	48	0.70 (0.52‐0.94)	8	0.44 (0.22‐0.88)
*P*_trend_		<0.001		<0.001		<0.001

Abbreviations: CI, confidence interval; DDD, defined daily dose; HR, hazard ratio.

a Using age as time metric in the models adjusted for race, family history of prostate cancer; baseline smoking status, body mass index, hypertension, hyperlipidemia, benign prostatic diseases, diabetes, chronic kidney disease, use of aspirin, angiotensin‐converting enzyme inhibitors, insulin, vitamin E/multivitamin, finasteride, metformin, testosterone supplement, selenium, prostate‐specific antigen level; as well as atherosclerotic cardiovascular disease and cumulative number of prostate‐specific antigen tests as time‐dependent variables.

b Based on an equivalent dose of 20‐mg simvastatin.

### 3.3 Association of statin use with risk of grade‐specific PCa

In PCa Gleason grade‐stratified analysis, statin use was associated with decreased risk of both low‐grade (aHR, 0.85; 95% CI, 0.74‐0.96) and high‐grade PCa (aHR, 0.54; 95% CI, 0.42‐0.69), compared with no statin use. The risk of low‐grade PCa decreased with both increasing duration (≥102 months: aHR, 0.39; 95% CI, 0.26‐0.60; *P*_trend_ < .001) and dose (≥616 DDDs: aHR, 0.70; 95% CI, 0.52‐0.94; *P*_trend_ < .001) of statin use. The protective association was only observed when statins were used for ≥30 months or ≥121 DDDs. A short‐term statin use was associated with an increased low‐grade PCa risk (≤10 months: aHR, 2.03; 95% CI, 1.73‐2.37), but this risk increase was not found in statin cumulative dose analysis (Table 2). For high‐grade PCa, a decreased trend in the risk with increasing duration and dose of stain use was also observed, but no risk increase was found with low‐duration or low‐dose group (Table 2).

In Gleason score‐stratified analysis, the statin use‐associated PCa risk reduction was higher for PCa with a higher Gleason score (<7: aHR, 0.85; 95% CI, 0.74‐0.96; 9‐10: aHR, 0.24; 95% CI, 0.11‐0.54) (Table 3).

**Table 3:** Association of statin use with risk of Gleason score‐specific prostate cancer

Gleason score of prostate cancer	No statin use	Statin use
<7		
No. of cases	1868	440
HR (95% CI)^a^	1.00	0.85 (0.74‐0.96)
7 (3 + 4)		
No. of cases	224	35
HR (95% CI)^a^	1.00	0.62 (0.43‐0.90)
7 (4 + 3)		
No. of cases	149	20
HR (95% CI)^a^	1.00	0.49 (0.29‐0.82)
8		
No. of cases	115	21
HR (95% CI)^a^	1.00	0.60 (0.37‐0.96)
9‐10		
No. of cases	97	7
HR (95% CI)^a^	1.00	0.24 (0.11‐0.54)

Abbreviations: CI, confidence interval; HR, hazard ratio.

a Using age as time metric in the models adjusted for race, family history of prostate cancer; baseline smoking status, body mass index, hypertension, hyperlipidemia, benign prostatic diseases, diabetes, chronic kidney disease, use of aspirin, angiotensin‐converting enzyme inhibitors, insulin, vitamin E/multivitamin, finasteride, metformin, testosterone supplement, selenium, prostate‐specific antigen level; as well as atherosclerotic cardiovascular disease and cumulative number of prostate‐specific antigen tests as time‐dependent variables.

### 3.4 Association of statin lipophilicity with PCa risk

When statins were categorized by lipophilicity, only lipophilic statins were associated with decreased PCa risk (overall PCa: aHR, 0.83; 95% CI, 0.72‐0.95; high‐grade PCa: aHR, 0.63; 95% CI, 0.48‐0.83), whereas no significant association was observed of only hydrophilic statins or both lipophilic and hydrophilic statins with PCa risk (Table 4).

**Table 4:** Association of statin lipophilicity with risk of overall and Gleason grade‐specific prostate cancer

Statin exposure	No. of sample	Overall		Gleason 2‐6		Gleason 7‐10	
		No. of cases	HR (95% CI)^a^	No. of cases	HR (95% CI)^a^	No. of cases	HR (95% CI)^a^
No use of statins	9226	2453	1.00	1868	1.00	585	1.00
Type of statins used							
Only lipophilic	3185	456	0.83 (0.72‐0.95)	384	0.86 (0.74‐1.01)	72	0.63 (0.48‐0.83)
Only hydrophilic	328	46	0.91 (0.63‐1.33)	40	1.04 (0.69‐1.57)	6	0.48 (0.20‐1.16)
Both lipophilic and hydrophilic	326	21	0.77 (0.49‐1.22)	16	0.80 (0.47‐1.36)	5	0.62 (0.25‐1.54)

Abbreviations: CI, confidence interval; HR, adjusted hazard ratio.

a Using age as time metric in the models adjusted for race, family history of prostate cancer; baseline smoking status, body mass index, hypertension, hyperlipidemia, benign prostatic diseases, diabetes, chronic kidney disease, use of aspirin, angiotensin‐converting enzyme inhibitors, insulin, vitamin E/multivitamin, finasteride, metformin, testosterone supplement, selenium, prostate‐specific antigen level; as well as atherosclerotic cardiovascular disease and cumulative number of prostate‐specific antigen tests as time‐dependent variables.

### 3.5 Sensitivity analyses

The statin use‐associated PCa risk reduction was only observed among those with baseline PSA ≥4 ng/mL (Table S2), but the risk reduction was consistent across subgroups of baseline CVD diagnosis (Table S3). Prescriptions of statins and aspirin were highly correlated (*χ* ^2^ = 4128.6, *P* < .001, agreement = 80.2%, (7448 + 3024)/13065). Compared with no use of statins or aspirin, use of only statins without aspirin was significantly associated with a risk reduction only in high‐grade PCa (Table S4).

## 4 DISCUSSION

Results of this large hospital‐based cohort study indicated that statin use might be associated with decreased risk of both low‐ and high‐Gleason grade PCa in a duration‐ and dose‐response mode. Of note, this statin‐related PCa risk reduction was observed only when statins had been used for a relatively longer duration and were more pronounced for PCa of a higher Gleason score. Lipophilic statins might have a better anti‐PCa effect than hydrophilic statins. These findings add to evidence to elucidate the association of statin use with PCa risk.

A common challenge in studying statin use and PCa risk was “healthy user bias.”^14^, ^15^, ^16^ Statin users, who might use health services more frequently than nonstatin users, possibly also have more opportunities for PSA screening and therefore have a decreased risk of high‐grade PCa.^18^, ^19^ If this explanation holds, the risk of low‐grade PCa may increase among statin users.^20^ But in our study, statin use was associated with decreased risk of both low‐ and high‐grade PCa. This indicates that the “healthy user bias” may not fully explain the observed association of statin use and PCa risk in our study.

In the current study, statin users had higher prevalence of comorbidities at baseline (Table 1). This is a problem that was also found in several other population‐/hospital‐based studies of statin use and PCa risk.^21^, ^22^ Elevated serum cholesterol levels and related comorbidities, for example CVD and hypertension, are known to promote PCa initiation.^23^, ^24^ Even so, we found a protective association of statin use with PCa risk after adjustment for comorbidities. In sensitivity analyses stratified by baseline CVD, the statin use‐related PCa risk reduction was consistent across those with and without CVD (Table S3). It indicates a low possibility that CVD history, as a major reason for statin prescription, had exaggerated the observed benefits of statin use.

The protective association of statin use with PCa risk is in line with in vitro studies. Mechanistically, statins reduce intracellular and serum cholesterol, thus may affect cell membrane organogenesis, steroidogenesis, and proliferation.^25^ Growth inhibition in prostate‐derived cell lines was indeed observed at clinically relevant statin concentrations.^26^, ^27^ In addition, to clarify the overall association of statin use on PCa risk, a further effort is needed to determine whether this association is duration‐/dose‐dependent. In our study, risks of both low‐ and high‐Gleason grade PCa were observed to decrease with increasing cumulative duration and cumulative dose of statin use (*P*_trend_ < .001), and significant PCa risk reduction was observed only when statins had been used for a relatively longer duration. This is in agreement with cell culture study which reported that only a long‐term statin exposure could induce cell apoptosis, G1 cell cycle arrest, autophagy, and degradation of androgen receptors.^28^ If statins impact PCa risk via the action on lipid metabolism, lipophilicity of statins may be important in determining their cancer‐preventive capability.^29^ In the current study, we only found a significant protective association of lipophilic statins with PCa risk. This is in line with observational and experimental studies^30^, ^31^ which reported a reduced number of colonies in clonogenic assays with lipophilic statins, but not with hydrophilic statins. But more studies are warranted to confirm this finding and elucidate involved mechanisms.

Our study has several strengths. First, this is one of the largest longitudinal studies to have studied statin use in relation to Gleason score‐specific PCa. With a long follow‐up period of 94 802 person‐years, we identified a substantial number of incident cancer cases. Our study was thus well‐powered to investigate the outcome of interest. Also, we linked various databases of demographics, lab tests, prescriptions, medical diagnoses, and others, enabling us to account for a number of potential key confounders, including indications of statin prescription, cumulative number of PSA testing and PSA levels. Meanwhile, several limitations are worth noting. In this study, data of statin use were extracted from medical records, thus misclassification of statin exposure resulting from prescription noncompliance or other statin sources was beyond our control. However, this potential exposure misclassification would likely dilute the HRs toward the null. Secondly, our study population was selected on the basis of urologic clinic visits rather than general population, thus we cannot rule out the possibility of selection bias. Although this potential bias may limit the generalizability of our findings, we believe using the noncancerous urologic patients as the without‐event group may have made the observed effect conservative. Also, although we were able to conduct a Gleason score‐specific analysis, the Gleason score was determined by prostate biopsy rather than radical prostatectomy specimen, thus the possibility of under‐sampling and under‐grading cannot be ruled out. Last, based on the observation that statin use (in binary format, yes vs no) was significantly associated with reduced risk of both low‐ and high‐grade PCa (Table 2), we speculate that the observed benefit of statin use cannot be fully due to “healthy users bias.” But for specific category of statin use duration, for example <10 month, we cannot rule out the possibility of “healthy user bias,” especially considering patients with a shorter statin use duration are at their early stage of lipid screening, thus they are more likely to be prescribed a PSA testing. But, no increased risk of low‐grade PCa was observed for low dose of statin use. Therefore, more studies are needed to replicate this finding and confirm involved mechanisms.

In conclusion, this study indicated that statin use might be associated with decreased risk of both low‐ and high‐Gleason grade PCa, particularly when statins had been used for a relatively longer duration. Also, the statin‐related PCa risk reduction was higher for PCa of a higher Gleason score, and lipophilic statins might act better than hydrophilic statins in PCa prevention. More studies are needed to confirm these findings.

## CONFLICT OF INTEREST

The authors declare no conflict of interest.

## AUTHOR CONTRIBUTIONS

KW and MP designed the study and conducted the main analysis. All authors participated in interpreting the results and drafting the manuscript. All the authors approved this final version of the manuscript.
