# Supplementary material for: Association of statin use with risk of Gleason score‐specific prostate cancer: A hospital‐based cohort study
Source: Cancer Med. 2019 Oct 8;8(17):7399–407. doi: 10.1002/cam4.2500 (PMC6885894; doi:10.1002/cam4.2500)
Supplement: Supplementary file 1 [file CAM4-8-7399-s001.docx]

**Supplementary Material**

Association of Statin Use with Risk of Gleason Score-Specific Prostate Cancer: A Hospital-Based Cohort Study

**Table of contents**

**Supplementary table s1**: ICD-9 codes of prostatic conditions used in this study

**Supplementary table s2**: Association of statin use with risk of overall and Gleason grade-specific prostate cancer, stratified by PSA level at baseline

**Supplementary table s3**: Association of statin use with risk of overall and Gleason grade-specific prostate cancer, stratified by atherosclerotic cardiovascular disease at baseline

**Supplementary table s4**: Association of statin and aspirin use with risk of overall and Gleason grade-specific prostate cancer Supplementary table s1. ICD-9 codes of prostatic conditions used in this study

| ICD-9 code | ICD-9 code description |
| --- | --- |
| 98.12 | Gonococcal prostatitis (acute) |
| 98.32 | Gonococcal prostatitis chronic |
| 131.03 | Trichomonal prostatitis |
| 185 | Malignant neoplasm of prostate |
| 222.2 | Benign neoplasm of prostate |
| 233.4 | Carcinoma in situ of prostate |
| 236.5 | Neoplasm of uncertain behavior of prostate |
| 600 | Hypertrophy (benign) of prostate without urinary obstruction and other lower urinary tract (luts) |
| 600.01 | Hypertrophy (benign) of prostate with urinary obstruction and other lower urinary tract symptoms (luts) |
| 600.1 | Nodular prostate without urinary obstruction |
| 600.11 | Nodular prostate with urinary obstruction |
| 600.2 | Benign localized hyperplasia of prostate without urinary obstruction and other lower urinary tract symptoms (luts) |
| 600.21 | Benign localized hyperplasia of prostate with urinary obstruction and other lower urinary tract symptoms (luts) |
| 600.3 | Cyst of prostate |
| 600.9 | Hyperplasia of prostate, unspecified, without urinary obstruction and other lower urinary symptoms (luts) |
| 600.91 | Hyperplasia of prostate, unspecified, with urinary obstruction and other lower urinary symptoms (luts) |
| 601 | Acute prostatitis |
| 601.1 | Chronic prostatitis |
| 601.2 | Abscess of prostate |
| 601.3 | Prostatocystitis |
| 601.4 | Prostatitis in diseases classified elsewhere |
| 601.8 | Other specified inflammatory diseases of prostate |
| 601.9 | Prostatitis unspecified |
| 602 | Calculus of prostate |
| 602.1 | Congestion or hemorrhage of prostate |
| 602.2 | Atrophy of prostate |
| 602.3 | Dysplasia of prostate |
| 602.8 | Other specified disorders of prostate |
| 602.9 | Unspecified disorder of prostate |
| 790.93 | Elevated prostate specific antigen (PSA) |

Supplementary table s2A. Association of statin use with risk of overall and Gleason grade-specific prostate cancer, stratified by PSA level at baseline

|  | Baseline PSA <4 ng/mL (n=7206) | | | | | |
| --- | --- | --- | --- | --- | --- | --- |
|  | Overall | | Gleason 2-6 | | Gleason 7-10 | |
| Statin exposure | NO. of cases | HR (95% CI)^*^ | NO. of cases | HR (95% CI)^*^ | NO. of cases | HR (95% CI)^*^ |
| No use of statins | 601 | 1.00 | 448 | 1.00 | 153 | 1.00 |
| Use of statins | 160 | 0.83 (0.67-1.02) | 121 | 0.85 (0.67-1.09) | 39 | 0.75 (0.51-1.12) |
| Cumulative duration, months |  |  |  |  |  |  |
| Quint 1: 1-10 | 41 | 1.99 (1.43-2.75) | 34 | 2.09 (1.46-3.01) | 7 | 1.53 (0.71-3.30) |
| Quint 2: 11-29 | 30 | 0.78 (0.54-1.14) | 25 | 0.85 (0.56-1.29) | 5 | 0.54 (0.22-1.32) |
| Quint 3: 30-60 | 31 | 0.58 (0.40-0.85) | 25 | 0.63 (0.41-0.95) | 6 | 0.44 (0.19-1.04) |
| Quint 4: 61-101 | 36 | 0.76 (0.53-1.09) | 26 | 0.76 (0.50-1.15) | 10 | 0.78 (0.39-1.54) |
| Quint 5: 102-239 | 22 | 0.61 (0.39-0.95) | 11 | 0.44 (0.23-0.82) | 11 | 1.07 (0.55-2.05) |
| *P* _trend_ |  | 0.001 |  | 0.001 |  | 0.269 |
| Cumulative dose, DDDs^†^ |  |  |  |  |  |  |
| Quint 1: 5-40 | 44 | 1.01 (0.72-1.41) | 34 | 1.06 (0.71-1.56) | 10 | 0.90 (0.47-1.72) |
| Quint 2: 41-120 | 54 | 1.01 (0.75-1.35) | 37 | 0.96 (0.67-1.38) | 17 | 1.09 (0.64-1.87) |
| Quint 3: 121-240 | 16 | 0.49 (0.29-0.81) | 14 | 0.57 (0.33-0.99) | 2 | 0.23 (0.06-0.92) |
| Quint 4: 241-615 | 20 | 0.57 (0.36-0.90) | 17 | 0.65 (0.39-1.08) | 3 | 0.32 (0.10-1.05) |
| Quint 5: 616-38750 | 26 | 0.89 (0.59-0.90) | 19 | 0.89 (0.55-1.46) | 7 | 0.91 (0.42-1.98) |
| *P* _trend_ |  | 0.015 |  | 0.068 |  | 0.070 |

Supplementary table s2B

|  | Baseline PSA ≥4 ng/mL (n=1287) | | | | | |
| --- | --- | --- | --- | --- | --- | --- |
|  | Overall | | Gleason 2-6 | | Gleason 7-10 | |
| Statin exposure | NO. of cases | HR (95% CI)^*^ | NO. of cases | HR (95% CI)^*^ | NO. of cases | HR (95% CI)^*^ |
| No use of statins | 474 | 1.00 | 303 | 1.00 | 171 | 1.00 |
| Use of statins | 67 | 0.63 (0.47-0.84) | 47 | 0.67 (0.47-0.96) | 20 | 0.47 (0.29-0.77) |
| Cumulative duration, months |  |  |  |  |  |  |
| Quint 1: 1-10 | 17 | 1.01 (0.61-1.68) | 13 | 1.11 (0.61-2.01) | 4 | 0.64 (0.23-1.79) |
| Quint 2: 11-29 | 19 | 0.74 (0.46-1.19) | 13 | 0.78 (0.43-1.39) | 6 | 0.61 (0.27-1.38) |
| Quint 3: 30-60 | 14 | 0.44 (0.26-0.76) | 9 | 0.44 (0.22-0.85) | 5 | 0.38 (0.16-0.93) |
| Quint 4: 61-101 | 11 | 0.55 (0.30-1.00) | 7 | 0.54 (0.26-1.14) | 4 | 0.48 (0.18-1.30) |
| Quint 5: 102-239 | 6 | 0.53 (0.24-1.19) | 5 | 0.70 (0.29-1.72) | 1 | 0.21 (0.03-1.44) |
| *P* _trend_ |  | <0.001 |  | 0.009 |  | 0.002 |
| Cumulative dose, DDDs^†^ |  |  |  |  |  |  |
| Quint 1: 5-40 | 22 | 0.70 (0.45-1.10) | 15 | 0.75 (0.43-1.30) | 7 | 0.56 (0.26-1.19) |
| Quint 2: 41-120 | 15 | 0.56 (0.32-0.96) | 11 | 0.62 (0.33-1.17) | 4 | 0.37 (0.13-1.04) |
| Quint 3: 121-240 | 10 | 0.56 (0.30-1.02) | 6 | 0.51 (0.23-1.13) | 4 | 0.52 (0.21-1.34) |
| Quint 4: 241-615 | 11 | 0.57 (0.31-1.07) | 7 | 0.55 (0.25-1.18) | 4 | 0.55 (0.19-1.54) |
| Quint 5: 616-38750 | 9 | 0.83 (0.42-1.65) | 8 | 1.09 (0.52-2.31) | 1 | 0.23 (0.03-1.81) |
| *P* _trend_ |  | 0.014 |  | 0.100 |  | 0.010 |

Supplementary table s2C

|  | Baseline PSA unknown (n=4572) | | | | | |
| --- | --- | --- | --- | --- | --- | --- |
|  | Overall | | Gleason 2-6 | | Gleason 7-10 | |
| Statin exposure | NO. of cases | HR (95% CI)^*^ | NO. of cases | HR (95% CI)^*^ | NO. of cases | HR (95% CI)^*^ |
| No use of statins | 1378 | 1.00 | 1117 | 1.00 | 261 | 1.00 |
| Use of statins | 296 | 0.86 (0.73-1.00) | 272 | 0.90 (0.76-1.07) | 24 | 0.41 (0.27-0.64) |
| Cumulative duration, months |  |  |  |  |  |  |
| Quint 1: 1-10 | 175 | 2.11 (1.77-2.51) | 164 | 2.23 (1.85-2.68) | 11 | 0.97 (0.52-1.81) |
| Quint 2: 11-29 | 77 | 0.78 (0.62-0.99) | 72 | 0.83 (0.65-1.06) | 5 | 0.32 (0.13-0.78) |
| Quint 3: 30-60 | 27 | 0.33 (0.22-0.48) | 21 | 0.29 (0.19-0.46) | 6 | 0.39 (0.17-0.88) |
| Quint 4: 61-101 | 10 | 0.17 (0.09-0.32) | 9 | 0.18 (0.09-0.35) | 1 | 0.09 (0.01-0.65) |
| Quint 5: 102-239 | 7 | 0.26 (0.12-0.54) | 6 | 0.25 (0.11-0.57) | 1 | 0.20 (0.03-1.41) |
| *P* _trend_ |  | <0.001 |  | <0.001 |  | <0.001 |
| Cumulative dose, DDDs^†^ |  |  |  |  |  |  |
| Quint 1: 5-40 | 105 | 1.07 (0.84-1.35) | 95 | 1.14 (0.89-1.47) | 10 | 0.56 (0.30-1.03) |
| Quint 2: 41-120 | 97 | 1.05 (0.82-1.35) | 88 | 1.08 (0.83-1.40) | 9 | 0.63 (0.31-1.30) |
| Quint 3: 121-240 | 48 | 0.83 (0.60-1.16) | 44 | 0.86 (0.61-1.22) | 4 | 0.38 (0.14-1.07) |
| Quint 4: 241-615 | 25 | 0.45 (0.30-0.67) | 24 | 0.48 (0.32-0.74) | 1 | 0.10 (0.02-0.73) |
| Quint 5: 616-38750 | 21 | 0.45 (0.29-0.72) | 21 | 0.51 (0.32-0.80) | 0 | - |
| *P* _trend_ |  | <0.001 |  | <0.001 |  | <.001 |

Abbreviations: HR, hazard ratio; CI, confidence interval; DDD, defined daily dose.

^*^Using age as time metric in the models adjusted for race, family history of prostate cancer; baseline smoking status, body mass index, hypertension, hyperlipidemia, benign prostatic diseases, diabetes, chronic kidney disease, use of aspirin, angiotensin-converting enzyme inhibitors, insulin, vitamin E/multivitamin, finasteride, metformin, testosterone supplement, selenium; as well as atherosclerotic cardiovascular disease and cumulative number of prostate-specific antigen tests as time-dependent variables.

^†^Based on an equivalent dose of 20 mg simvastatin.

Supplementary table s3A. Association of statin use with risk of overall and Gleason grade-specific prostate cancer, stratified by atherosclerotic cardiovascular disease at baseline

|  | With atherosclerotic cardiovascular disease at baseline (n=2579) | | | | | |
| --- | --- | --- | --- | --- | --- | --- |
|  | Overall | | Gleason 2-6 | | Gleason 7-10 | |
| Statin exposure | NO. of cases | HR (95% CI)^*^ | NO. of cases | HR (95% CI)^*^ | NO. of cases | HR (95% CI)^*^ |
| No use of statins | 353 | 1.00 | 287 | 1.00 | 66 | 1.00 |
| Use of statins | 204 | 0.75 (0.61-0.92) | 172 | 0.79 (0.63-0.99) | 32 | 0.52 (0.33-0.82) |
| Cumulative duration, months |  |  |  |  |  |  |
| Quint 1: 1-10 | 79 | 1.58 (1.22-2.04) | 72 | 1.66 (1.26-2.19) | 7 | 0.92 (0.41-2.08) |
| Quint 2: 11-29 | 50 | 0.73 (0.54-0.99) | 43 | 0.76 (0.55-1.06) | 7 | 0.50 (0.23-1.08) |
| Quint 3: 30-60 | 34 | 0.49 (0.35-0.71) | 26 | 0.47 (0.31-0.72) | 8 | 0.51 (0.25-1.04) |
| Quint 4: 61-101 | 25 | 0.45 (0.30-0.70) | 20 | 0.46 (0.28-0.74) | 5 | 0.37 (0.14-0.93) |
| Quint 5: 102-239 | 16 | 0.42 (0.24-0.70) | 11 | 0.39 (0.20-0.73) | 5 | 0.46 (0.18-1.21) |
| *P* _trend_ |  | <0.001 |  | <0.001 |  | 0.003 |
| Cumulative dose, DDDs^†^ |  |  |  |  |  |  |
| Quint 1: 5-40 | 61 | 0.89 (0.66-1.20) | 50 | 0.93 (0.67-1.30) | 11 | 0.75 (0.38-1.46) |
| Quint 2: 41-120 | 67 | 0.91 (0.68-1.23) | 60 | 0.99 (0.72-1.37) | 7 | 0.43 (0.18-1.04) |
| Quint 3: 121-240 | 30 | 0.65 (0.44-0.97) | 23 | 0.63 (0.40-0.98) | 7 | 0.61 (0.28-1.30) |
| Quint 4: 241-615 | 24 | 0.51 (0.33-0.79) | 21 | 0.56 (0.35-0.89) | 3 | 0.28 (0.09-0.89) |
| Quint 5: 616-38750 | 22 | 0.53 (0.34-0.83) | 18 | 0.54 (0.33-0.89) | 4 | 0.43 (0.17-1.12) |
| *P* _trend_ |  | 0.001 |  | <0.001 |  | 0.002 |

Supplementary table s3B

|  | Without atherosclerotic cardiovascular disease at baseline (n=10486) | | | | | |
| --- | --- | --- | --- | --- | --- | --- |
|  | Overall | | Gleason 2-6 | | Gleason 7-10 | |
| Statin Exposure | NO. of cases | HR (95% CI)^*^ | NO. of cases | HR (95% CI)^*^ | NO. of cases | HR (95% CI)^*^ |
| No use of statins | 2100 | 1.00 | 1581 | 1.00 | 519 | 1.00 |
| Use of statins | 319 | 0.80 (0.70-0.91) | 268 | 0.86 (0.75-0.99) | 51 | 0.47 (0.35-0.63) |
| Cumulative duration, months |  |  |  |  |  |  |
| Quint 1: 1-10 | 154 | 2.04 (1.72-2.40) | 139 | 2.23 (1.87-2.66) | 15 | 0.88 (0.52-1.48) |
| Quint 2: 11-29 | 76 | 0.75 (0.60-0.95) | 67 | 0.83 (0.65-1.06) | 9 | 0.35 (0.18-0.67) |
| Quint 3: 30-60 | 38 | 0.37 (0.27-0.51) | 29 | 0.36 (0.25-0.53) | 9 | 0.30 (0.15-0.58) |
| Quint 4: 61-101 | 32 | 0.43 (0.30-0.61) | 22 | 0.39 (0.25-0.60) | 10 | 0.46 (0.24-0.87) |
| Quint 5: 102-239 | 19 | 0.47 (0.30-0.73) | 11 | 0.36 (0.20-0.66) | 8 | 0.67 (0.33-1.36) |
| *P* _trend_ |  | <0.001 |  | <0.001 |  | <0.001 |
| Cumulative dose, DDDs^†^ |  |  |  |  |  |  |
| Quint 1: 5-40 | 110 | 0.99 (0.80-1.24) | 94 | 1.12 (0.89-1.42) | 16 | 0.51 (0.30-0.84) |
| Quint 2: 41-120 | 99 | 0.90 (0.72-1.12) | 76 | 0.88 (0.68-1.13) | 23 | 0.81 (0.52-1.26) |
| Quint 3: 121-240 | 44 | 0.70 (0.51-0.96) | 41 | 0.82 (0.59-1.13) | 3 | 0.18 (0.06-0.56) |
| Quint 4: 241-615 | 32 | 0.47 (0.33-0.68) | 27 | 0.51 (0.34-0.75) | 5 | 0.25 (0.10-0.61) |
| Quint 5: 616-38750 | 34 | 0.74 (0.52-1.05) | 30 | 0.81 (0.56-1.18) | 4 | 0.36 (0.13-0.96) |
| *P* _trend_ |  | <0.001 |  | 0.001 |  | <0.001 |

Abbreviations: HR, hazard ratio; CI, confidence interval; DDD, defined daily dose.

^*^Using age as time metric in the models adjusted for race, family history of prostate cancer; baseline smoking status, body mass index, hypertension, hyperlipidemia, benign prostatic diseases, diabetes, chronic kidney disease, use of aspirin, angiotensin-converting enzyme inhibitors, insulin, vitamin E/multivitamin, finasteride, metformin, testosterone supplement, selenium, prostate-specific antigen level; as well as cumulative number of prostate-specific antigen tests as time-dependent variable.

^†^Based on an equivalent dose of 20 mg simvastatin.

Supplementary table s4. Association of statin and aspirin use with risk of overall and Gleason grade-specific prostate cancer

|  |  | Overall | | Gleason 2-6 | | Gleason 7-10 | |
| --- | --- | --- | --- | --- | --- | --- | --- |
| Exposure | No. of sample | NO. of cases | HR (95% CI)^*^ | NO. of cases | HR (95% CI)^*^ | NO. of cases | HR (95% CI)^*^ |
| No use of statins or aspirin | 7448 | 2104 | 1.00 | 1587 | 1.00 | 517 | 1.00 |
| Use of only statins | 815 | 174 | 1.08 (0.90-1.29) | 161 | 1.26 (1.05-1.52) | 13 | 0.30 (0.17-0.54) |
| Use of only aspirin | 1778 | 349 | 0.74 (0.66-0.84) | 281 | 0.75 (0.65-0.86) | 68 | 0.63 (0.48-0.83) |
| Use of both statins and aspirin | 3024 | 349 | 0.60 (0.53-0.70) | 279 | 0.60 (0.51-0.70) | 70 | 0.54 (0.40-0.72) |

Abbreviations: HR, hazard ratio; CI, confidence interval.

^*^Using age as time metric in the models adjusted for race, family history of prostate cancer; baseline smoking status, body mass index, hypertension, hyperlipidemia, benign prostatic diseases, diabetes, chronic kidney disease, use of angiotensin-converting enzyme inhibitors, insulin, vitamin E/multivitamin, finasteride, metformin, testosterone supplement, selenium, prostate-specific antigen level; as well as atherosclerotic cardiovascular disease and cumulative number of prostate-specific antigen tests as time-dependent variables.
